# Assessing the Incidence of Head Trauma in Australian Mixed Martial Arts: A Retrospective Analysis of Fight Outcomes

**DOI:** 10.1177/19417381241263332

**Published:** 2024-08-02

**Authors:** Daniel A. Brown, Garret Gross

**Affiliations:** † School of Health Sciences and Social Work, Nathan, Griffith University, Brisbane, Queensland, Australia; ‡ Griffith Sports Science, Griffith University, Gold Coast, Queensland, Australia

**Keywords:** chronic traumatic encephalopathy, combat sports, concussion, head injuries, MMA

## Abstract

**Background:**

Mixed martial arts (MMA) is experiencing a surge in popularity in Australia. Previous research has suggested knockout (KO) and technical knockout (TKO) are frequent outcomes during competition, raising concern about the brain health of athletes. This study aims to describe fight outcomes in Australian MMA and to explore differences in fight-ending outcomes between male and female athletes, amateur and professional competition, and different weight classes.

**Hypothesis:**

There is no difference in the incidence of KO/TKO between level of competition, sex, and weight class.

**Study Design:**

Descriptive epidemiology study.

**Level of Evidence:**

Level 3.

**Methods:**

Retrospective analysis of 143 Australian MMA events from 2020 to 2023 was conducted using video replay to assess fight outcomes between sex and level of competition. Binary logistic regression analysis was used to determine relationships between weight class and KO/TKO fight outcomes.

**Results:**

Male competition (34%) had a significantly greater number of KO/TKO secondary to head strikes fight outcomes compared with female competition (23%) (*P* = 0.01). The KO/TKO rate secondary to head strikes for amateur and professional male competition was 16.6 and 18.7 per 100 athlete-exposures (AEs), respectively. The amateur and professional female rate was 12.6 and 7.4 per 100 AEs, respectively. Amateur male light heavyweight and heavyweight, and professional male heavyweight were at greater odds of a KO or TKO compared with other weight classes in their equivalent level of competition.

**Conclusion:**

There is a sex and professional level disparity in the incidence of fight-ending head trauma in Australian MMA. The study findings highlight the urgent need for targeted safety protocols and medical oversight, particularly for men in heavier weight classes.

**Clinical Relevance:**

This study highlights the need for enhanced safety protocols and medical oversight in Australian MMA, particularly for male athletes in heavier weight divisions.

The sport of mixed martial arts (MMA) has surged in global popularity.^3^ As a full-contact combat sport, combining elements from grappling and striking sports, the objective in MMA is to effectively damage and control an opponent.^15^ Governed by a unified set of rules, an athlete may be victorious through strikes (punches, kicks, elbows, and knees), submission (sportive choke and joint-lock techniques), or judge’s decision.^20^ Unlike many sports, MMA rules allow for intentional contact to the head, exposing athletes to repetitive subconcussive and concussive impact, possibly leading to neurological injury.^1,6,7^ Several medical associations, including the Western Australian branch of the Australian Medical Association, have previously voiced apprehension and called for the restriction or ban of MMA competition.^16^ Many concerns raised are in relation to the brain health of athletes who participate in MMA.^15,16^

In MMA competition, knockout (KO) and technical knockout (TKO) are a common occurrence.^15^ A KO refers to when an athlete is unconscious or disorientated, and a TKO refers to an athlete who is unable to defend themselves intelligently or logically.^15,21^ Previous studies have reported the incidence of concussion defined by KO and TKO secondary to head strikes to be as high as 15.9 per 100 athlete-exposures (AEs) in professional MMA competitions.^10,15^ It is commonly accepted that a KO meets the criteria of concussion; however, TKO is not as clear.^15^ A TKO may be a result of repetitive head impacts, strikes to the body, injury, and doctor or coach stoppage. In respect to athletes who sustain a TKO secondary to head strikes, concussion may be a resultant event.^11,15^

Understanding fight-ending outcomes and associated potential health risks is important to help guide medical professional triage to provide safe and immediate care for athletes.^26^ Health-related consequences have been associated with repetitive concussive and subconcussive strikes to the head in combat athletes, including those who participate in MMA.^2,5,19^ Despite the increased awareness regarding head trauma-related health consequences, recent concern has also been raised regarding the safety of sportive chokes. Overall, sportive chokes appear to be safe; however, in rare cases, ischemic stroke and cervical arterial dissection may occur.^24,25^ Although complications are reported to be rare, it is important that medical professionals are aware of the risk of sequelae of sportive chokes during competitive MMA bouts.

Despite previous research exploring fight outcomes in MMA competition, the majority has involved professional athletes.^11,15,17^ To the best of our knowledge, no study has investigated fight outcome in MMA competition in Australia. Furthermore, concerns have been raised regarding the exposure risk of amateur athletes participating in MMA in Australia.^19^ Currently, there is a limited understanding of frequent fight-ending outcomes in male and female amateur competition; however, potential differences in fight outcome related to head trauma may exist.^23^ Therefore, the purpose of the present study is to describe fight outcomes in Australian MMA competition and examine the prevalence of fight-ending head trauma. The secondary aim was to explore differences in fight-ending outcomes between male and female athletes and between amateur and professional competition.

## Methods

### Data Collection

The present study is a retrospective cohort investigation of competition between male and female athletes, amateur and professional competition, and different weight classes in MMA events held in Australia from July 1, 2020 to July 31, 2023. This study was approved by the host institution’s Human Research Ethics Committee (HREC reference No: 2023/558). Events included in the study were limited to those governed by Australian-based MMA organizations. Events governed by international organizations were excluded. Events without available video replay of fight outcomes were excluded. Data for the study were collected from specialized publicly available websites (https://www.tapology.com/ and https://www.sherdog.com/) to obtain fight information, including athletes, sex, weight class, level of competition (amateur or professional), and fight outcome.^11,15^ Videos of events were obtained from Australian-based MMA organizations’ websites and subsequently reviewed by both authors. The use of video replay served to verify fight details. Similar strategies have been employed in previous studies concerning data collection for MMA competition outcomes.^11,15^

### Level of Competition

Classification of fights was used to determine the level of competition. In the present study, professional athletes compete under the Unified Rules of MMA. Athletes were categorized as “amateur” if they competed in amateur B-Class or C-Class bouts. In addition to the Unified Rules of MMA, amateur (B-Class) bouts in Australia do not allow for elbow strikes, rotational kicks, spinal rotation locks, or upkicks to the head. In addition to B-Class rules, bouts that are competed in the C-Class classification are excluded from striking to the head of a ground opponent, rotational punches, or knee strikes to the head, and often use 10-ounce (283.5 g) gloves rather than 4-ounce (113.4 g), and occasional use of shin pads. Title bouts were typically held over 5 rounds, with nontitle bouts consisting of 3 rounds. A typical professional round was scheduled for 5 minutes, and amateur rounds scheduled for 3 minutes.

### Fight Outcome

The present study considered various fight outcomes, including KO, TKO, submission, decision (unanimous, split or draw), disqualification, and no contest. A KO is indicated by a fighter’s inability to defend, typically due to a loss of consciousness or disorientation.^15,23^ This was accepted as the definition for KO in the present study. A KO has previously been accepted to meet the criteria of concussion.^10,15^ A TKO is declared when the referee, physician, or coach believes the fighter has ceased intelligently defending against their opponent.^15,23^ Video analysis was used to identify TKOs that were secondary to head strikes. For the present study, TKO secondary to head strikes was used as a surrogate for concussion.^15^ AE, as defined in previous studies, referred to an athlete being exposed to the potential of incurring injury (KO, or TKO secondary to head strikes) during a single bout (1 bout resulted in 2 AEs).^10,11^ In addition, video analysis was used to verify submission technique used to finish a bout. Submissions were categorized as “sportive choke,” “joint lock,” or “verbal.”

### Weight Class

The weight classes for male fighters included flyweight (≤125 lb [≤57 kg]), bantamweight (125-135 lb [57-61 kg]), featherweight (136-145 lb [62-66 kg]), lightweight (146-155 lb [66-70 kg]), welterweight (156-170 lb [71-77 kg]), middleweight (171-185 lb [78-84 kg]), light heavyweight (186-205 lb [84-93 kg]), heavyweight (206-265 lb [93-120 kg]), and super heavyweight (>265 lb [>120 kg]). Female weight classes consisted of strawweight (≤115 lb [≤52 kg]), flyweight (116-125 lb [53-57 kg]), bantamweight (125-135 lb [57-61 kg]), featherweight (136-145 lb [62-66 kg]), and lightweight (146-155 lb [66-70 kg]).

### Statistical Analysis

Descriptive statistics were used to determine fight outcome, sex of athlete, and weight class. Male and female divisions were divided into level of competition (amateur and professional divisions). Pearson’s chi-squared (*χ*^2^) tests were used to examine differences between male and female competition and the level of competition. To align with previous research, fight-ending outcomes were normalized to 100 AEs.^10^ Binary logistic regression analyses examined the relationship between weight classes and the occurrence of “KO or TKO secondary to head strikes” for both sexes. For men, the lightweight class served as the reference, as per previous research.^11,15^ Due to limited bouts in the male super heavyweight and strawweight class, they were excluded from the binary logistic regression analysis. Due to the sample size across all female divisions and classes, athletes from both levels of competition were combined. Athletes were then separated into “light” (strawweight and flyweight) and “heavy” (bantamweight, featherweight, lightweight) categories. All data were analyzed using SPSS Version 29.0 (IBM).

## Results

### Description of Fight Events in Australian MMA

During the study period, 194 events were identified and occurred across 6 states and 1 territory in Australia. Video record was available for 143 (74%) events from 24 promotional organizations and were included in the study for analysis. A total of 1473 bouts were reviewed, involving 1476 unique athletes. A significant majority of competition was in male divisions, which accounted for 92.3% (n = 1359) of the total dataset. Competition at the professional level accounted for 24.2% of all fights analyzed. The distribution of athletes across all weight classes is reported in Table 1. The mean male amateur and professional bout lasted 2.24 ± 0.98 rounds and 2.13 ± 1.07 rounds, respectively. Female amateur and professional competition lasted 2.13 ± 1.02 rounds and 2.11 ± 0.93 rounds, respectively. Across all MMA competition in Australia, the KO or TKO secondary to head strikes rate was 16.7 per 100 AE. Male competition (34%) had a significantly greater proportion of fights ending via KO or TKO secondary to head strikes compared with female competition (23%), *χ*^2^ (1, n = 1473) = 6.31, *P* = 0.01.

**Table 1.** Distribution of athletes in each weight class

	Amateur Male	Professional Male	Total Male	Amateur Female	Professional Female	Total Female
Strawweight, n (%)	1 (<1)	0 (0)	1 (<1)	15 (17)	6 (22)	21 (18)
Flyweight, n (%)	63 (6)	22 (7)	85 (6)	30 (35)	11 (41)	41 (36)
Bantamweight, n (%)	120 (12)	33 (10)	153 (11)	18 (21)	5 (19)	23 (20)
Featherweight, n (%)	217 (21)	68 (21)	285 (21)	14 (16)	4 (15)	18 (16)
Lightweight, n (%)	202 (20)	70 (21)	272 (20)	7 (8)	1 (4)	8 (7)
Welterweight, n (%)	220 (21)	69 (21)	289 (21)	2 (2)		2 (2)
Middleweight, n (%)	104 (10)	39 (12)	143 (11)			
Light heavyweight, n (%)	49 (5)	7 (2)	56 (4)			
Heavyweight, n (%)	52 (5)	22 (7)	74 (5)	1 (1)		1 (1)
Super heavyweight, n (%)	1 (<1)		1 (<1)			
Total, n	880	258	1138	87	27	114

### Examination of Fight Outcome in Australian MMA Events

#### Male Competition

The most frequent outcome across all male bouts was decision (38%). A KO was recorded in 8.9% of all male bouts. The frequency of amateur and professional male fight-ending outcomes is displayed in Table 2. The proportion of “KO or TKO secondary to head strikes” did not differ between amateur and professional fights, *χ*^2^ (1, n = 1359) = 1.99, *P* = 0.16. The number of AEs for male amateur and professionals were 2058 and 660, respectively. For amateur male fight outcomes, the rate of KO was 3.8 per 100 AE, and for “KO or TKO secondary to head strikes” was 16.6 per 100 AE. In professional male fights, the rate of KO was 6.2 per 100 AE, and “KO or TKO secondary to head strikes” was 18.7 per 100 AE.

**Table 2.** Comparison of male amateur and professional fight outcomes in Australia

	Amateur	Professional	Total
KO, n (%)	80 (7.8)	41 (12.4)	121 (8.9)
TKO, n (%)	290 (28.2)	99 (30.0)	389 (28.6)
Submission, n (%)	241 (23.4)	85 (25.8)	326 (24.0)
Decision, n (%)	415 (40.3)	103 (31.2)	518 (38.1)
Other, n (%)^*a*^	3 (0.3)	2 (0.6)	5 (0.5)
Total, n (%)	1029 (75.7)	330 (24.3)	1359 (100)
KO or TKO secondary to head strikes, n (%)	343 (33.3)	124 (37.6)	389 (34.2)

KO, knockout; TKO, technical knockout.

a No contest and disqualification.

The most frequent fight-ending submission category was “sportive choke” in both amateur (n = 204, 84.6%) and professional (n = 79, 92.9%) in male competition. The rear naked choke was the most frequent fight-ending submission in both amateur (n = 127, 12.3%) and professional (n = 47, 14.2%) competition. The verbal submissions were related to musculoskeletal injury that resulted in an athlete verbally submitting from the contest.

**Figure 1.**
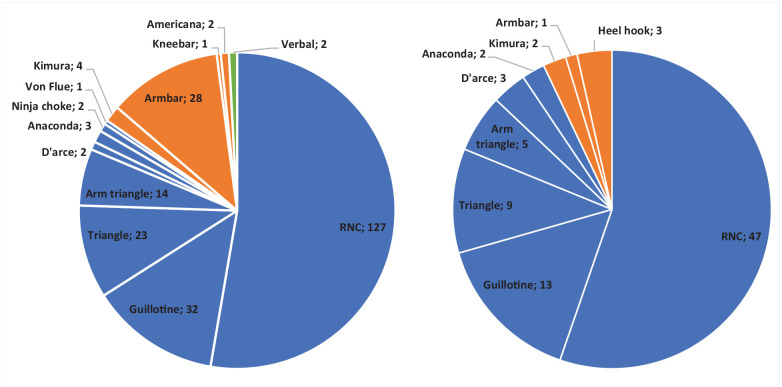
Breakdown of submission type and frequency (n) in (a) amateur and (b) professional male competition. Blue represents “sportive choke” submissions, orange represents joint-lock submissions, green represents verbal submission. RNC, rear naked choke.

The fight-ending submission frequencies are displayed in Figure 1.

#### Female Competition

The most frequent fight-ending outcome in female MMA competition was decision (58%). A TKO or submission occurred in 24% and 18% of all female fights, respectively. The fight outcomes across amateur and professional female fights are shown in Table 3. The proportion of KO or TKO secondary to head strikes did not differ between amateur and professional fights, *χ*^2^ (1, n = 114) = 1.2, *P* = 0.26. The number of AEs in the study for female amateur and professionals were 174 and 54, respectively. The KO or TKO secondary to head strikes rate was 12.6 per 100 AE for amateur and 7.4 per 100 AE for professional female fights. Combined, female MMA athletes had a KO or TKO secondary to head strikes rate of 11.6 per 100 AE.

**Table 3.** Comparison of female amateur and professional fight outcomes in Australia

	Amateur	Professional	Total
KO, n (%)	0 (0)	1 (4)	1 (1)
TKO, n (%)	24 (28)	3 (11)	27 (24)
Submission, n (%)	13 (15)	7 (26)	20 (18)
Decision, n (%)	50 (58)	16 (59)	66 (58)
Other, n (%)^*a*^	0 (0)	0 (0)	0 (0)
Total, n (%)	87 (79)	27 (21)	114 (100)
KO or TKO secondary to head strikes, n (%)	22 (25)	4 (15)	26 (23)

KO, knockout; TKO, technical knockout.

a No contest and disqualification.

**Figure 2.**
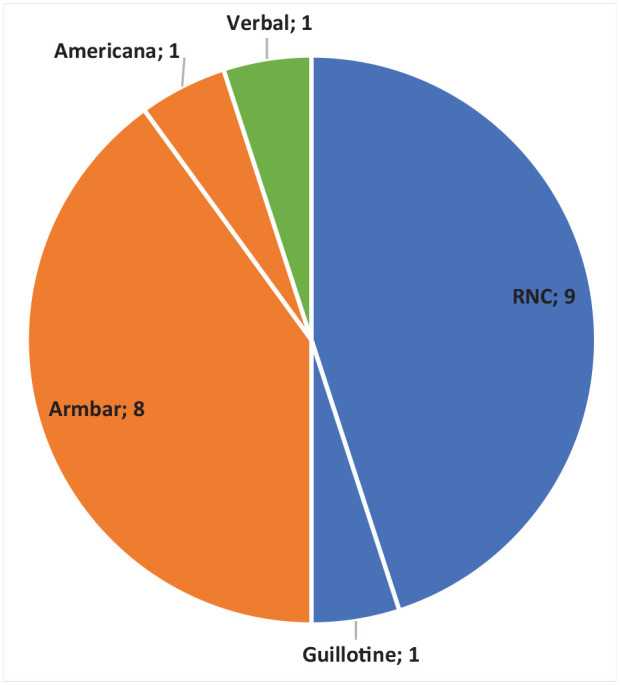
Fight-ending submission frequency in all female competition (n). Blue represents “sportive choke” submissions, orange represents joint-lock submissions, green represents verbal submission. RNC, rear naked choke.

The most frequent fight-ending submission in female competitive MMA was “sportive choke” (n = 10, 50%). Figure 2 displays the frequency (n) of fight-ending submissions across all female competitions. The armbar, a “joint-lock” technique, was the most frequently submission in amateur female competition (n = 6, 46%), followed by the rear naked choke (n = 4, 31%). For professional female competition, the rear naked choke technique was the most frequent submission outcome (n = 5, 71%), followed by the arm bar (n = 2, 29%).

#### Weight Class

Across all male MMA bouts, the heavyweight division had the highest proportion of bouts ending in KO or TKO secondary to head strikes (n = 43, 58%, 29.1 per 100 AE), followed by light heavyweight (n = 28, 50%, 25 per 100 AE), welterweight (n = 110, 38%, 19.0 per 100 AE), middleweight (n = 53, 37%, 18.5 per 100 AE), lightweight (n = 82, 30%, 15.1 per 100 AE), and featherweight (n = 92, 32%, 16.1 per 100 AE). Flyweight (n = 20, 24%, 11.7 per 100 AE) and bantamweight (n = 39, 26%, 12.7 per 100 AE) had the lowest proportion of KO or TKO secondary to head strikes outcomes. Table 4 displays the odds of KO or TKO secondary to head strikes outcome between weight classes for amateur and professional male bouts. Due to the low sample across the 7 weight classes, the female group was separated into “lighter” and “heavier” categories. In the “lighter” weight class, a KO or TKO secondary to head strikes occurred in 18% of fights, at a rate of 9.0 per 100 AE. A similar result occurred in 28% of fights in the “heavier” category, at a rate of 14.2 per 100 AE. Overall, there was a 10% difference in the occurrence of KO or TKO secondary to head strikes between the 2 female categories. However, there was no statistical difference in the odds of a KO or TKO secondary to head strikes outcome between the categories, odds ratio (95% CI) = 0.983, *P* > 0.05.

**Table 4.** ORs for KO or TKO secondary to head strikes according to weight class for male amateur and professional fights

	Amateur		Professional	
	OR (95% CI)	*P*	OR (95% CI)	*P*
Lightweight	1.0		1.0	
Flyweight	0.60 (0.30-1.19)	0.14	1.10 (0.39-3.01)	0.87
Bantamweight	0.67 (0.40-1.13)	0.14	1.33 (0.56-3.20)	0.52
Featherweight	1.10 (0.73-1.67)	0.65	1.12 (0.54-2.30)	0.77
Welterweight	1.43 (0.95-2.14)	0.09	1.41 (0.70-2.86)	0.34
Middleweight	1.22 (0.74-2.02)	0.43	1.80 (0.80-4.01)	0.16
Light heavyweight	2.22 (1.18-4.20)	0.01	3.11 (0.64-15.13)	0.16
Heavyweight	2.70 (1.45-5.03)	<0.05	5.00 (1.78-14.04)	<0.01

KO, knockout; OR, odds ratio; TKO, technical knockout.

## Discussion

### Competition in Australian MMA

The present study highlights the prominence of MMA in Australia. Between 2020 and 2023, we identified 194 events identified held across 6 states and 1 territory. The substantial availability of video records for many of these MMA events not only highlights the growing documentation and accessibility of the sport but may have potential healthcare benefits. Video analysis has been employed in sports to aid in the identification of injuries, including concussion.^13^ While the presence of a ringside physician is paramount during combat sporting events, video review could further support healthcare professionals in injury identification and subsequent clinical decision-making.

Furthermore, our findings suggest that MMA is a male-dominated sport in Australia, accounting for over 90% of all competition. The disparity between male and female MMA participation in Australia aligns with the historical representation of combat sports being dominated by male athletes.^18^ Previous research has suggested that participation in combat sports may increase female health and confidence.^18^ Thus, further research is required to explore the context to female participation in MMA in Australia and investigate strategies for recruitment and retention in sport.

## Head Trauma in Australian MMA—Male Athletes

The current study is the first to examine and describe the fight-ending outcome frequencies in Australian MMA competition. The overall KO incidence in these competitions was 4 per 100 AE, accounting for 8% of all competitions. Differentiating between genders and competition levels, the KO incidence for male amateur bouts was marginally lower at 3.8 per 100 AE, compared with the professional division at 6.2 per 100 AE. Both groups align with previously reported rates in professional MMA, which range from 1.6 to 6.7 per 100 AE.^8,11,15,22^ To further explore head trauma exposure, we amalgamated KO data with TKO outcomes that were attributed to repetitive head strikes. The combined incidence of KO and TKO secondary to head strikes were 16.6 and 18.7 per 100 AE for amateur and professional male competition, respectively. These rates observed for both male amateur and professional competition slightly exceed the previously reported figures in professional MMA, ranging between 15.4 and 15.9 per 100 AE.^11,15^

## Head Trauma in Australian MMA—Female Athletes

In professional female competition, a single KO was observed, while amateur competition had none. The results of this study suggest a lower KO rate in female MMA competition compared with male competition in Australia. Interestingly, these findings contrast from previous research that found no discernible difference in KO rates between male and female competition.^17^ The findings of the present study may reflect the relatively small sample size in female competition compared with male competition. When combining KO and TKO secondary to head strikes, we observed rates of 7.4 and 11.6 per 100 AE for amateur and professional competition, respectively. These results exceed previously reported rates for professional female MMA competition of 6.9 per 100 AE.^11^

## Head Trauma in Weight Classes

Through binary logistic regression analysis, with lightweight set as the reference, we found that the heavyweight division had higher odds of a fight stopping via KO or TKO secondary to head strikes in both male amateur and professional competition. Furthermore, the male amateur light heavyweight division also showed increased odds of KO or TKO secondary to head strikes. These findings have similarities to previous research, which observed greater odds of KO and TKO due to head strikes in the heavier divisions.^11,15^

Due to the constrained sample size of female competitors, we grouped athletes into “lighter” and “heavier” divisions. Interestingly, there was no significant difference in the odds of KO and TKO secondary to head strikes between these female divisions. While acknowledging methodological and sample variations, our findings in the female divisions contrast from with earlier studies that indicated a greater risk for KO and TKO for athletes in heavier divisions.^11^

## Submission Outcomes

In the male divisions, submissions accounted for 23% of amateur bouts and 25% of professional bouts. For women, 15% of amateur bouts ended in submission, while the percentage increased to 26% in professional bouts. These results present a contrast to previous research, which observed a submission outcome in 17% of professional male bouts and 21% in professional female bouts.^12^ Furthermore, sportive choke, particularly via rear naked choke, was the predominate submission technique in both amateur and professional male competition, as well as professional female competition. This observation is consistent with previous research investigating both professional male and female competition.^12^ For amateur female competition, we observed a preference for joint locks as the primary submission type for victory. The selection of submission type across different competition divisions may reflect the perceived effectiveness of certain techniques or the emphasis placed on them during training. These insights could prove valuable for athletes and coaches when devising training and competition strategies.

## Clinical Relevance

After a competitive MMA fight, athletes are routinely assessed by a qualified ring-side physician. The physician’s role is to screen for injury and provide medical advice. The Association of Ringside Physicians recommends combat sport athletes who lose via KO or TKO be medically suspended from competition and sparring for a set period, with the minimum timeframe of 30 days.^21^ This recommendation mirrors many requirements for athletes participating in collision sport in Australian, including rugby league, rugby union, and Australian Rules Football. However, despite the recommendation for medical suspension, approximately 60% of athletes will return to sport, including the participation of sparring, within 48 hours of a concussion and while symptomatic.^4,5,14^ Furthermore, in lieu of medical recommendations, athletes may elect to compete in an alternative organization or combat sport, including boxing and Muay Thai, before physiological or clinical recovery has been achieved. The loophole to return to competition not only heightens the risk of secondary concussion but also raises concern for the potential for catastrophic lifelong injuries. For this reason, it is a necessity for immediate attention and intervention to safeguard the wellbeing of the athletes.

With the rising popularity of MMA in both amateur and professional divisions in Australia, there is an expected increase in sport-related concussions. Proactive prevention strategies, including policy changes and educational initiatives, should be prioritized to guarantee athletes receive optimal care. A unified medical evaluation system and record-keeping across combat sports in the country is paramount. One suggestion is the use of a concussion passport, to maintain a historical record of concussion and monitor health throughout an athlete’s career.^9,27^ Implementing a national policy that collates KO, TKO, and medical suspensions could significantly reduce the chance of an athlete’s premature return to competition.^15^ Furthermore, educating coaches and athletes can enhance concussion awareness, encourage the reporting of concussion symptoms, and potentially lead to adjustments in training routines, including altered sparring session frequencies.

In addition, there are growing clinical concerns about the safety of sportive chokes. Recent literature has highlighted cases where sportive chokes led to severe complications, including cervical artery dissection and ischemic stroke.^24,25^ While such incidences are extremely rare, their potential long-term health implications cannot be ignored. Given the prevalent use of sportive chokes in Australian MMA competition, it is a necessity for heightened awareness among healthcare professionals, especially ringside and emergency physicians.

## Limitations

There are limitations to the current study. The relatively small proportion of female division competitions analyzed significantly limits the generalizability of findings specific to female athletes.

While TKO secondary to head strikes suggest the possibility of a concussion, it is not definitive evidence of one.^15^ Moreover, by analyzing only fight outcomes, potential concussions sustained by winning fighters are likely overlooked, likely leading to an underestimation of the true concussion incidence. The reliance on video records for analysis means that any event without a recorded video was excluded, leading to potential selection bias. Even with video analysis, the determination of fight outcomes was based on the clinical judgement of the reviewer, which may vary. In the absence of postfight medical evaluation records, the exact severity of injuries remains uncertain. It is also important to note that the findings of the current study may not be generalizable to other combat sports or MMA competitions. We did not separate amateur bouts in “B” and “C” class.

## Conclusion

The current study offers valuable insight into the landscape of MMA competition in Australia, emphasizing the prevalence of head trauma and sportive chokes. While MMA continues to grow in popularity, the findings emphasize the critical need for enhanced safety measures, comprehensive medical evaluations, and consistent record-keeping across combat sports. The differences in male and female competition, coupled with the risks associated with early return to competition after concussion, highlight areas that warrant further investigation.
